# Innovative Fusion Strategy for MEMS Redundant-IMU Exploiting Custom 3D Components †

**DOI:** 10.3390/s23052508

**Published:** 2023-02-24

**Authors:** Giorgio de Alteriis, Alessia Teresa Silvestri, Claudia Conte, Verdiana Bottino, Enzo Caputo, Antonino Squillace, Domenico Accardo, Rosario Schiano Lo Moriello

**Affiliations:** 1 Department of Industrial Engineering, University of Naples Federico II, Piazzale Tecchio 80, 80125 Naples, Italy; 2 Department of Chemical, Materials and Production Engineering, University of Naples Federico II, Piazzale Tecchio 80, 80125 Naples, Italy

**Keywords:** redundant-IMU, multi-sensors, Allan variance, data fusion, weighted average, additive manufacturing

## Abstract

In recent years, the overall performances of inertial Micro-Electro Mechanical Sensors (MEMSs) exhibited substantial improvements to values very close or similar to so-called tactical-grade sensors. However, due to their high costs, numerous researchers are currently focusing on the performance enhancement of cheap consumer-grade MEMS inertial sensors for all those applications (as an example, small unmanned aerial vehicles, UAVs), where cost effectiveness is a relevant request; the use of redundancy proves to be a feasible method for this purpose. In this regard, the authors propose, hereinafter, a suitable strategy aimed at fusing raw measurements provided by multiple inertial sensors mounted on a 3D-printed structure. In particular, accelerations and angular rates measured by the sensors are averaged according to weights associated with the results of an Allan variance approach; the lower the noise figure of the sensors, the greater their weight on the final averaged values. On the other hand, possible effects on the measurements due to the use of a 3D structure in reinforced ONYX (a material capable of providing better mechanical specifications for avionic applications with respect to other solutions for additive manufacturing) were evaluated. The performance of a prototype implementing the considered strategy is compared with that of a tactical-grade inertial measurement unit in stationary conditions, exhibiting differences as low as 0.3 degrees in heading measurements. Moreover, the reinforced ONYX structure does not significantly affect the measured values in terms of both thermal and magnetic field while assuring better mechanical characteristics with respect to other 3D printing materials, thanks to a tensile strength of about 250 MPa and a specific stacking sequence of continuous fibers. Finally, a test conducted on an actual UAV highlights performance very close to that of a reference unit, with root-mean-square error in heading measurements as low as 0.3 degrees in observation intervals up to 140 s.

## 1. Introduction

In the last decade, the market for Unmanned Aerial Vehicles (UAVs, commonly referred to as drones) has experienced rapid growth, mainly because of the substantial cost reduction and technological improvement associated with avionic systems and components, as also highlighted in the preliminary research [1]. In fact, compared to a helicopter or a fixed-wing aircraft, Unmanned Aircraft Systems (UASs), usually referred to as drones, exhibit reduced dimensions, increased autonomy, and a much lower cost; these are the main reasons why they represent a cost-effective solution to adopt in various fields, such as sensing, maintenance, infrastructure operations, precision agriculture, entertainment, and surveillance [2,3,4,5,6].

The increased efficiency and cost effectiveness of the drones are also associated with the capability of the onboard integration of several sensors at the same time, including high-resolution cameras, thermal cameras, and Micro-Electro Mechanical Sensors (MEMSs). [2,7,8,9,10,11]. In addition to the offered capabilities of reaching sufficient accuracy for most applications, MEMSs are able to overcome many of the drawbacks associated with most of today’s navigation units (which tend to be large, heavy, and expensive) thanks to their size, weight, and reduced cost [12]. Indeed, since navigation parameters constitute the main factors that can strongly affect the UAS unit’s performance, particular attention must be paid to MEMS inertial sensors; these are typically classified through bias instability and random walk, the two parameters that mainly characterize both accelerometer and gyroscope performance and also classify MEMS for the target applications [13,14,15]. Typically, gyroscopes with a bias instability lower than 1deg/h are referred to as “tactical-grade”. These sensors, whose cost is in the thousands of euros, are able to measure the earth rate and estimate the heading angle without the need to integrate other sensors. On the other hand, the more cost-effective “consumer-grade’’ sensors exhibit lower power consumption, but they are also characterized by lower performance [8,16].

Despite the numerous advantages associated with MEMS inertial sensors, drawbacks must be considered as well. Indeed, because of their reduced size, they are highly sensitive to environmental changes; in addition, the unpredictability of random noises affecting the measurements makes the error compensation procedures more complex, also limiting the sensor’s applicability [17,18]. An increasing bias drift showing non-linear characteristics is the main factor to be taken into account; such behavior demonstrates that the accuracy level ensured by MEMS IMUs is remarkable at high rates, while both angular velocity and acceleration data are easily degraded in the long term. This is the reason why a navigation system commonly relies on sensor fusion techniques; in particular, accelerometer, gyroscope, and magnetometer measurements are collected along with the Global Navigation Satellite System (GNSS) output, and then a Kalman filter algorithm that uses different inputs is implemented to estimate attitude and position, also minimizing the influence of stochastic errors [14,19,20]. Among these, one of the most relevant environmental factors causing MEMS performance’s degradation is represented by temperature fluctuations, since the change in thermal properties of the MEMS gyroscope materials determines a modification to their geometrical microstructure, affecting the performance of the sensor directly. Specifically, temperature shifts lead to a change in the main factors influencing the sensor output, i.e., Young’s modulus and the thermal expansion coefficients characterizing the materials, of which the gyroscope is realized [11].

Despite the Kalman filtering approach demonstrating its validity for error compensation, because of the above-mentioned issues, correction techniques have become increasingly complicated, and Inertial Measurement Units (IMUs) able to collect high-quality data are still relatively expensive; anyway, new innovative and accurate solutions, also more adaptable to environmental changes, are expected to be developed as the use of these instruments becomes more commonplace [7,17,20,21,22,23].

A suitable solution that can be proposed to overcome the limitations associated with MEMSs is the adoption of a redundant configuration; in fact, numerous studies have demonstrated that exploiting redundant geometrical forms allows one, theoretically, to decrease the global bias in each sensing unit [24], having the expansion of their field of applications as a result. Further, researchers have pointed out that integrating multiple IMUs on the same system offers the possibility to identify and isolate sensor failures, enabling easier error mitigation operations and so increasing both the system’s accuracy and robustness [18]. In particular, the method typically adopted to evaluate the sensor noise was based on Allan variance, according to the IEEE standard, determining the noise parameters of interest from the specific characteristic point, as described in [25].

At present, a specific and optimal procedure to combine the outputs of the sensors of which a redundant IMU is composed has not been fully defined, paving the way for different adaptable solutions; overall, not only the complete implementation of an Inertial Navigation System (INS) should be considered but also the sensor support structure and case, i.e., the drone payload, must be taken into account, with the aim to respect the weight, dimension, and robustness requirements. In this regard, an effective way to keep the MEMS advantages of smaller size and light weight was found in additive manufacturing technology (commonly known as 3D printing), a new technology used to customize industrial requests, which has allowed for the realization of a suitable structure, also exhibiting remarkable properties, where the most commonly used material is Polylactic Acid (PLA). Particularly, the technology used in this work, called Fused Filament Fabrication (FFF), combines fused deposition modeling with a second extruder (print head), depositing a continuous filament of reinforcing material. The base material used for this study, i.e., the ONYX, is patented by the Markforged^TM^ and consists of a combination of Nylon and chopped Carbon Fiber; when printed alone, this composition allows it to exhibit excellent mechanical properties, such as high strength, toughness, and chemical resistance, and it can also be reinforced with continuous fibers in order to yield aluminum-strength parts [26,27]. These properties, in addition to temperature resistance and fireproofing, are the main reasons permitting this innovative material to be successfully adopted in some aerospace applications, where the supporting structure has to pass stringent procedures in order to gain the specific certification [28].

In the present work, stemming from the preliminary research shown in [1], the authors propose an innovative strategy to fuse the acceleration, angular velocity, and magnetic field measurements obtained from a redundant IMU, having it mounted on a custom 3D structure realized with FFF technology. In order to gain performance close to that of a tactical-grade sensor or a certified Attitude and Heading Reference System (AHRS), the mechanical properties of additive manufacturing that are suitable for Unmanned Aerial Systems will be exploited and investigated, and a weighted average evaluated through the noise parameters obtained from the Allan variance will be adopted as well with the aim to obtain remarkable performance from a redundant configuration of low-cost MEMSs.

The paper is organized as follows: the proposed method and the implementation of the redundant-IMU prototype are described in Section 2, while in Section 3, the obtained results are presented as advantages introduced by the proposed fusion strategy, effect of the material adopted on measurement output, and then overall performance reached in attitude estimation, before drawing the conclusions in Section 4.

## 2. Proposed Method

**Figure 1:**
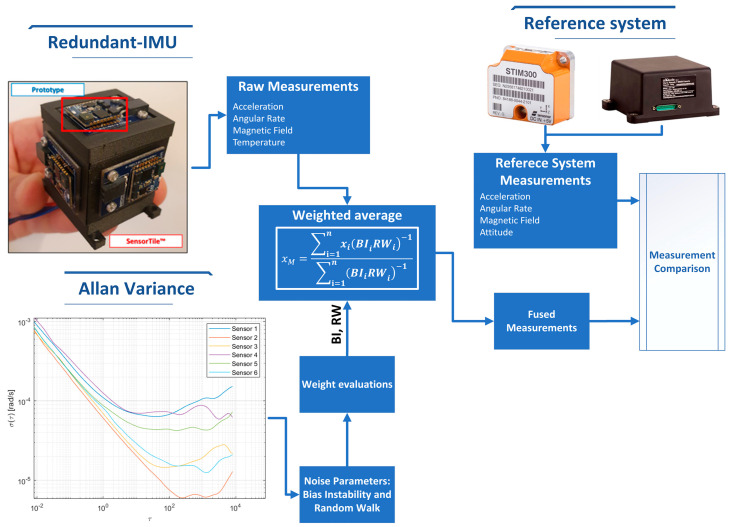
Proposed method exploiting the noise parameters and weighted average.

The proposed solution is based on the use of a redundant configuration of low-cost inertial sensors that ensure a remarkable performance increase, as described in [24]; in this work, the authors evaluated a method to fuse the acceleration, angular velocity, and magnetic field measurements of a redundant IMU made via FFF technology with the aim to improve the overall performance introduced by redundancy, and to test the effect of the material on the sensor measurements in such a way as to investigate any adverse effect on inertial and magnetic field measurements. In fact, the electronic component cloud suffers from errors due to the case, such as temperature and soft/hard iron effects. In particular, as shown in Figure 1, a redundant prototype was realized (5 × 5 × 5 cm^3^); then, the noise parameters, i.e., Bias Instability (BI) and Random Walk (RW), are evaluated by means of the Allan variance. The measurements of acceleration, angular velocity, and magnetic field are first refined by means of a weighted average capable of improving the reliability of the measured values. Finally, the results obtained in terms of acceleration and angular velocity are compared with a tactical-grade MEMS, while the magnetic field measurements and attitude estimates are compared with a certified AHRS, where both are adopted as measurement reference systems. Furthermore, the well-known temperature dependencies of a MEMS, as described in [29], have led to the evaluation of the internal temperature, which could affect the measures, with the aim to ensure the system performance also with the Carbon-Fiber-reinforced ONYX.

### 2.1. Hardware Architecture

**Figure 2:**
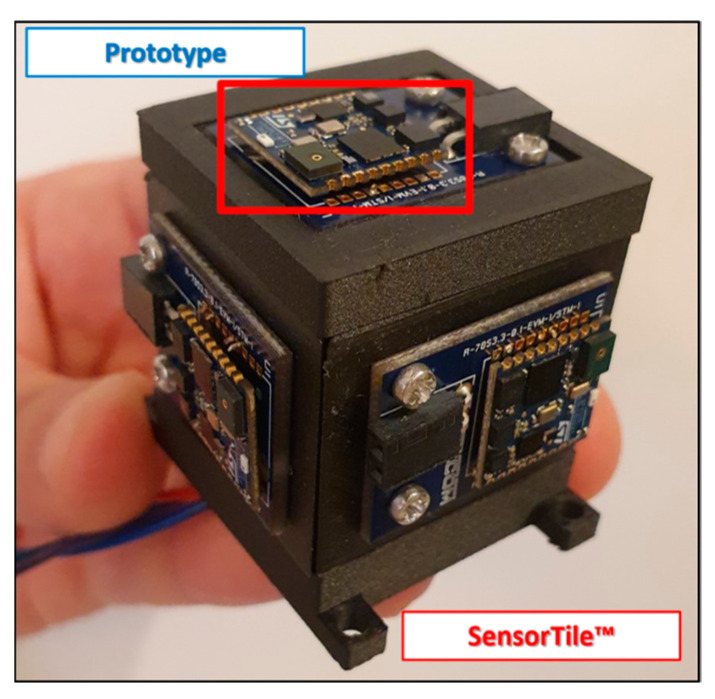
Multi-IMU redundant prototype made with reinforced ONYX.

Based on their previous experience, the authors chose a redundant multi-sensor IMU consisting of a triaxial set of low-cost and consumer-grade accelerometers, gyroscopes, and magnetometers in a cubic configuration, designed by the authors and manufactured by the FFF process using the reinforced ONYX material to obtain the advantages of robustness and light weight (Figure 2).

The sensor board used was the SensorTile^TM^, which is placed on each side of the prototype. There are six boards in total, consisting of a three-axis accelerometer, a three-axis gyroscope, and a three-axis magnetometer. The cubic configuration obtained is only 5 × 5 cm^2^ for each side. The nominal values of noise parameters are given in Table 1 and Table 2 in terms of Bias Instability (BI), Velocity Random Walk (VRW), and Angular Random Walk (ARW) for the accelerometer and gyroscope, respectively [24].

The SensorTiles are connected to a microcontroller from STMicroelectronics^TM^ that collects data from each sensor and stores the raw data on a microSD card or sends them to a Personal Computer; a full description of the system can be found in [24].

The STIM300 tactical-grade sensor from SensoNor^TM^ was chosen as a reference for the acceleration and angular velocity measurements; the noise parameters are listed in Table 3 [30], while the certified AHRS, called Axitude-Ax1 [31], is adopted as a reference system for magnetic field measurements and attitude estimates.

**Figure 3:**
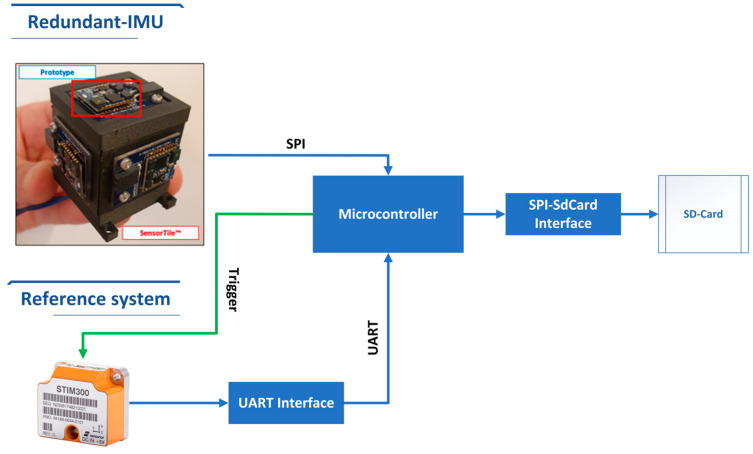
Measurement setup architectures for the prototype and STIM300.

The measurement setup architecture for the comparison between the prototype and the STIM300 is shown in Figure 3, where a microcontroller, i.e., STM32F446RE from STMicroelectronics^TM^, acquires data from the redundant IMU via an SPI (Serial Peripheral Interface) protocol, while from the STIM300 via the UART (Universal Asynchronous Receiver–Transmitter) protocol. For this purpose, an interface between the RS422 and UART protocols was realized with the adoption of dual differential drivers and receivers (SN75C1167 from Texas Instruments). Moreover, to synchronize the two systems, the STIM300 data are sampled through an external trigger provided by the microcontroller, which acquires data from the UART by means of a received interrupt on the serial port. The STIM300 is set to acquire samples at 2kHz and is triggered at 125Hz, thus limiting the mean delay between request and sampling of the measured quantities to 250 μs; the obtained value complies with the time constraints of navigation algorithms for this vehicle category for most of the applications. Finally, the acquired data are stored on a microSD-card that is connected to the microcontroller through an SPI-SdCard adaptor.

### 2.2. Noise Parameter Evaluation

The dependence of the quality of the state estimates on parameters defining the noise conditions of the sensor outputs is known to be one of the most important problems related to Kalman filtering. In order to adequately determine their values, different approaches can be followed. Based on their previous experience [24] and considering that the Allan Variance approach is widely adopted for noise parameter evaluations, the authors used Allan variance to obtain the desired values.

The Allan variance is first proposed to overcome problems associated with evaluating the standard deviation in an increasing sequence of collected data. Although Allan variance was originally used for oscillator frequency applications, it can also be used with inertial sensors to highlight and distinguish noise conditions added to the signal of interest [25]. Allan variance works successfully under two main conditions, i.e., the signal of interest remains constant during the measurements, and the noise average is zero for long-term recordings.

From an operational point of view, the Allan variance for a given cluster time *τ* is defined as half the time average of the squares of the differences between sensor outputs given by *τ* Equation (1).


(1) $$\sigma^{2}\left(\tau \right)=\frac{1}{2}\langle {\left(s\left(t-\tau \right)-s\left(t\right)\right)}^{2}\rangle$$

The bias instability and random walk that are associated with a flicker noise and white noise, respectively, are obtained according to the one-way relationship between the Allan variance and the two-side Power Spectral Density (PSD) of the noise parameters for the characteristic parameters of interest, equal to (referred to as Equation (2) and Equation (3), respectively):

(2) $$\sigma^{2}\left(\tau \right)=\frac{2BI^{2}\ln2}{\pi }$$

(3) $$\sigma^{2}\left(\tau \right)=\frac{RW^{2}}{\tau }$$

where $\sigma^{2}\left(\tau \right)$ is the Allan variance at time *τ*, *BI* is the bias instability coefficient, and *RW* is the random walk coefficient. The values are obtained from the curve portions with a slope equal to 0 and −1/2, respectively.

According to the IEEE standard [25], the test duration should be sufficient to determine performance characteristics where the record length must be about three orders of magnitude with respect to the frequency of interest, i.e., flicker noise. If prefiltering of the raw data is required to minimize the effect of quantization, the test results will be different than if prefiltering is not used. PSDs are useful to isolate and identify specific frequency components that may be present in the gyroscope and accelerometer output. Moreover, the temperature must remain constant throughout the duration of the acquisition, which affects the measurements. The data acquisition stage is a crucial aspect of obtaining meaningful results from the Allan variance. The first problem is to choose a proper sampling frequency and record length. The expected time range of bias instability for this low-cost MEMS is about 1 s and 100 s, so the geometrical mean was considered, as suggested in the standard, and the minimum sample period must be equal to 10$\sqrt{10}$ s and the minimum record length must be equal to 10^3^$\sqrt{10}$ s; this way, to suitably appreciate the desired parameters, the raw accelerometer and gyroscope measurements are acquired in a time window of 48 h at 125 Hz without any filtering stage. Moreover, the acquisition is performed in a climatic chamber to keep the temperature constant at 25 ± 1 °C, and data are stored in the microSD-card.

### 2.3. Weighted Average

The weighted average is defined as the sum of each measure multiplied by the associated weight divided by the sum of the weight. Higher weight is associated with the most accurate measurement with a narrow distribution, according to Equation (4). The information about the correct weight in this application is retrieved from the Allan variance results.

(4) $$x_{M}=\frac{\sum_{i=1}^{n}x_{i}{\left(BI_{i}RW_{i}\right)}^{-1}}{\sum_{i=1}^{n}{\left(BI_{i}RW_{i}\right)}^{-1}}\;$$

where $x_{M}$ is the weighted average for the three axes, $x_{i}$ is the accelerometer raw measurements, $BI_{i}$ and $RW_{i}$ are the Bias Instability and Random Walk values associated with each sensor, and n is the number of sensors adopted. The weighted average angular rates are also evaluated according to Equation (4).

The raw measurements from the redundant IMU are collected for 48 h; then, the weight of each sensor is evaluated by means of the Allan variance and according to the IEEE standard. The weighted average is applied to fuse the obtained raw measurements, where the weights were the reciprocals of BI and RW values retrieved from the characteristic points. Finally, the results in terms of acceleration and angular rate are compared with the reference system, as shown in the Section 3.

### 2.4. Fused Filament Fabrication

The Markforged™ “Mark Two” was the exploited composite 3D-printing machine. As mentioned in the Introduction, two materials were employed: Onyx as matrix (a combination strand of Nylon and short Carbon Fibers, hereinafter CF) and continuous Carbon Fibers as reinforcement.

Short CFs not only act as a reinforcement and improve the mechanical performance of the composite blend but also change the behavior of the material during the cooling phase by causing less thermal deformation. As a result, the dimensions of the additively manufactured parts match, as closely as possible, the model produced in CAD. Compared to bare nylon, onyx is about 3.5-times more resistant, has a higher hardness, and an HDT (Heat Deflection Temperature) of 140 °C. Concerning the continuous CF, with conventional composite manufacturing technologies, accurate alignment of the continuous fibers is still difficult and time and cost intensive. The additive CFF process can solve and overcome this issue in the production of composites since, with this technology, the alignment of the continuous fibers can be controlled and arranged in the direction of printing. This is important since the type and amount of reinforcement determine the final properties of the parts, and as with conventional processes, the highest strength and stiffness are achieved with continuous-fiber-reinforced composites [28,32].

The CAD (Computer-Aided Design) model and the relative STL file were produced using SolidWorks^®^ software. The 3D-printed part is a cube composed of 6 panels; during the CAD phase, the panels were designed with specific features, such as discontinuities and overhangs, to be combined after the printing process. With the same approach, the support structure was custom designed and printed to minimize the weight keeping high mechanical strength (Figure 2). The STL files were imported in EIGER, the associated software to Markforged, where it is possible to select several process parameters of interest, including the layer thickness, the layer material, and the orientation of the fibers.

**Figure 4:**
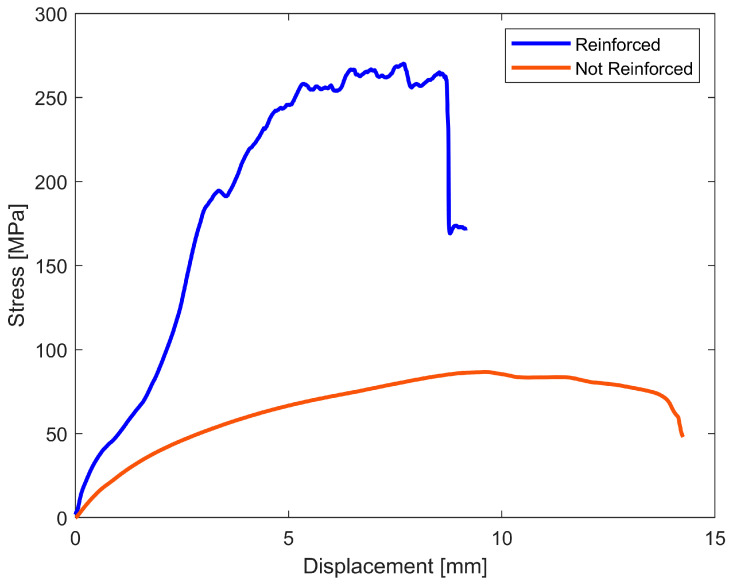
Stress versus displacement curves: comparison between reinforced and non-reinforced specimen.

Each panel is made up of 16 layers characterized by a layer thickness of 0.125 mm. The first and last 4 layers are in only Onyx, and the central 8 layers contain the continuous CFs with a stacking sequence that allows one to achieve the best mechanical performance: 0°, 45°, 90°, and 135°, as illustrated in Figure 4. The curves illustrate the comparison between specimens printed in only Onyx and those printed with the above-mentioned configuration. It is possible to note that with the latter, the mechanical strength is 3-times higher than the former. Moreover, in the reinforced sample, the initial damage did not lead to the collapse of the entire structure, but the peaks and valleys of the curve represent the progressive failure of the layers and/or part of continuous fibers. In this way, with correct monitoring or maintenance, it is possible to predict the failure of the structure, preserving the integrity of the sensor.

**Figure 5:**
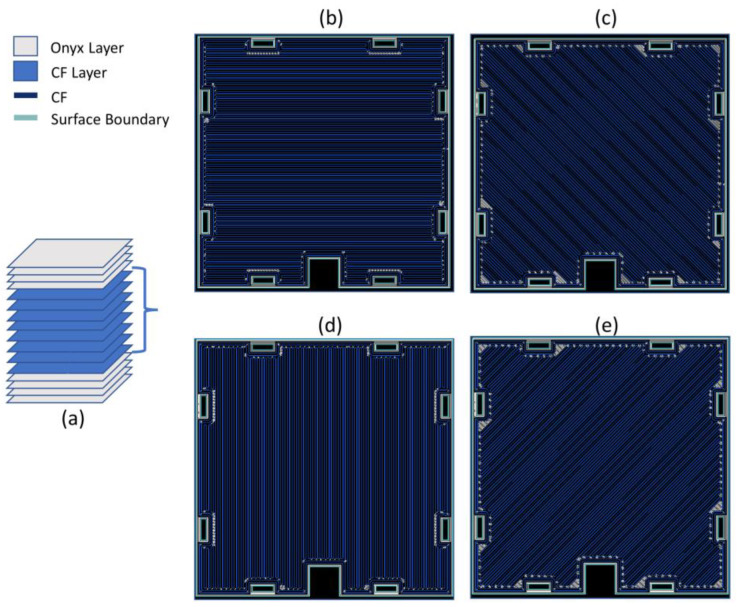
Schematic representations of the panel and the stacking sequence. (**a**) single panel composed of 16 layers; layers reinforced with continuous Carbon Fibers printed at (**b**) 0°, (**c**) 45°, (**d**) 90°, and (**e**) 135°.

Figure 5 shows the configuration of the panel: in grey, the former and latter group of layers in Onyx; in blue, the one composed of continuous CF, specifically an image of each layer printed with continuous CF is reported where the blue lines represent the CF and the light-green lines represent the surface boundaries made in Onyx in order to reach a good surface finish.

This is the 3D component adopted; in particular, it is a composite, and it is composed of ONYX as a matrix and Continuous Carbon Fibers as reinforcement. The choice of this configuration is based on the better mechanical properties performed by the reinforced component, highlighted in the Stress–Displacement graph, where the blue line is the component with the CF and the red without them. Moreover, this configuration was chosen considering that the temperature could lead to a softening or to slow degradation of the material and so the future performance of the case it is made of. For example, PLA, which is the most commonly used material in Fused Deposition Modeling, has a lower heat deflation temperature (50 °C) in comparison to Onyx (145 °C) [26,33] and a lower starting temperature degradation, 270 °C [34], against 400 °C for Onyx [26].

The support structure design is already evaluated in previous work [24], where a cubic configuration of a low-cost MEMS was adopted. The new prototype case is realized with the aim to meet the typical avionics requirements that require a sensor enclosure for protection. Moreover, the use of additive manufacturing technology allows one to achieve weight and overall dimension reductions but enhanced mechanical performance, which could be crucial aspects in this and other fields of application.

## 3. Results

In this research, three main aspects were evaluated: (i) the first analysis highlights the advantages introduced by the weighted average where the performance reached is assessed by means of a comparison between the proposed solutions and a tactical-grade MEMS; (ii) the second investigations aim to evaluate the adoption of the reinforced ONYX material and the effect it has on measurements, not only for the structure but also as a case for the navigation system, i.e., accelerometer, gyroscope, and magnetometer. In particular, the temperature and IMU measurements are acquired and compared with the same geometrical box made from PLA; (iii) finally, to assess the overall performance reached, a comparison between the prototype and a certified AHRS was evaluated in two conditions, i.e., static and dynamic.

### 3.1. Weighted Average Improvements

**Figure 6:**
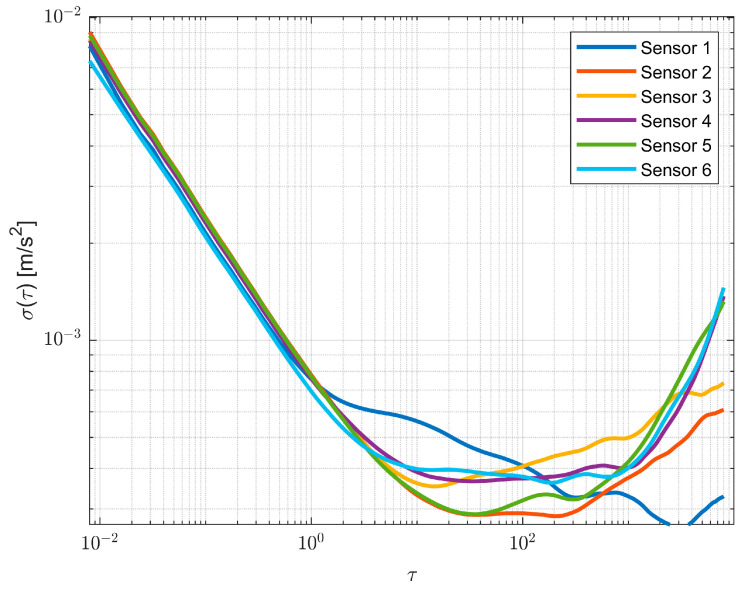
Accelerometer Allan variance evaluation for each sensor referred to the Z-axis.

The tests are carried out in static conditions and compared with a reference system, where both sensors are placed on the same plane and the sampling time is set to 125 Hz; in this way, the acquired data are obtained in the same operative conditions for both systems. This test aimed to preliminarily verify the enhancement effect of the weighted average associated with Allan Variance, i.e., noise parameters, as a fusion algorithm of the raw measurements of each sensing axis, both for the accelerometer and gyroscope. The Allan results obtained are shown in Figure 6, and for the sake of clarity, Z-axis of the accelerometer was selected, but similar results are obtained from the other axes and sensors.

The BI and RW values for each sensor are evaluated according to Equation (2) from the curve portion that presents a slope equal to 0 and −1/2. Widely different performance is highlighted from the Allan results. In fact, in this application, consumer-grade sensors were adopted; due to the fabrication process, the bias instability could also be much different, not only from the typical datasheet value but also from each sensor.

**Figure 7:**
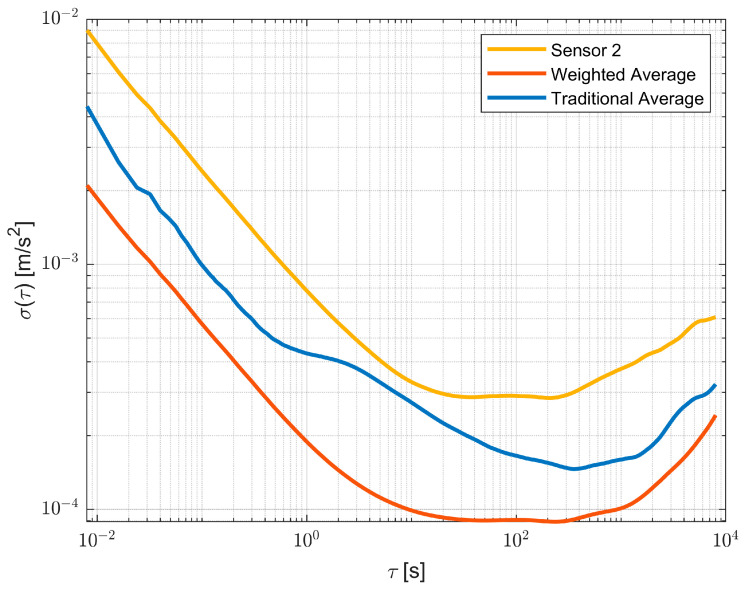
Allan variance comparison between one sensor in orange, traditional average in blue, and weighted average in red.

Once the BI and RW values are obtained, these are used according to Equation (4) as weights to obtain the fused raw measurements of the multi-IMU platform. The BI and RW results obtained with the proposed approach are reported in Table 4, while Figure 7 shows the improvements and the accuracy reached by the proposed method in terms of Bias Instability and Random Walk, where a comparison between one single sensor is selected as the best case (in orange), with raw measurements fused by means of the traditional average (in blue) and the proposed approach (in red); therefore, the performance improvements can be appreciated.

The performance reached by the proposed method is evaluated as a static comparison under the same environmental conditions with the raw measurements of the reference system and the weighted average in terms of Root Mean Square Error (RMSE), mean value, and standard deviation of the differences between the prototype and the reference. The results obtained are reported in Table 5, which highlights the improvements introduced by the proposed method, which were found to be very close to the reference value.

**Figure 8:**
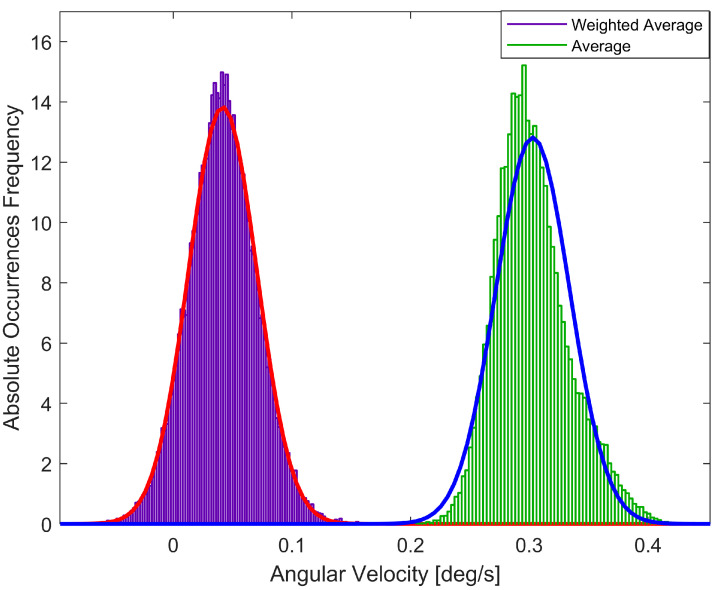
PDF comparison between the gyroscope raw measurements evaluated as weighted average and average referred to the Z-axis.

Moreover, in order to highlight the advantages of the proposed solution, in Figure 8, the weighted average and traditional average value Probability Density Function (PDF) were evaluated. The raw data are collected in five minutes at 125Hz.

The analysis carried out (for the sake of brevity, only the gyroscope Z-axis is shown, but similar results are obtained for the other axis and the accelerometer) pointed out that the raw measurements evaluated according to the proposed method have an improved Gaussian trend with respect to the raw measurement average. This effect has a remarkable impact in a navigation application where a Kalman-Filter-based approach is adopted. In fact, these sensors are typically used in a data fusion algorithm that estimates the attitude, position, and velocity of a vehicle, and the Kalman Filter, prediction and correction stages operate best for Gaussian noise.

### 3.2. Reinforced ONYX Material: Effect on Measurements

Whereas architectures similar to the one proposed have not been investigated at present, namely, the use of previously described additive structures with consumer-type inertial sensors, two particularly interesting aspects related to the nature of these sensors are observed: thermal drift and hard and soft iron phenomena. In fact, MEMSs are strongly affected by temperature with regard to accelerometers and gyroscopes, while magnetometers are affected by hard and soft iron phenomena. For this purpose, the structure on which they are mounted is preliminarily evaluated to see whether sensor measurements are affected by the type of material.

**Figure 9:**
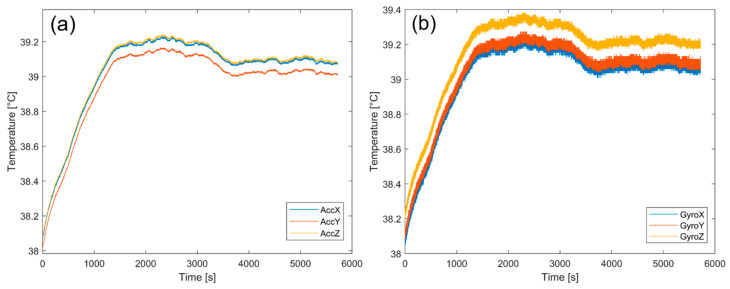
(**a**) Average internal temperature of the six prototype accelerometers. (**b**) Average internal temperature of the six prototype gyroscopes.

The first analysis is conducted by observing the internal temperature trends of both acceleration and angular velocity sensors over time; the results obtained are shown in Figure 9a,b respectively, for the accelerometer and the gyroscope along the three axes, where the temperature values are obtained as the average of the six sensors in order to observe the overall behavior of the prototype for a time of about 100 min. It is observed that after the transient regime lasting about 15 min, both sensors reach temperatures of 39 °C and 39.2 °C for the accelerometer and the gyroscope, respectively, with a variation of about 0.2 °C throughout the duration of the acquisition. Therefore, the material used did not significantly affect the sensors and, thus, the measurements; in fact, no significant thermal delta was observed.

**Figure 10:**
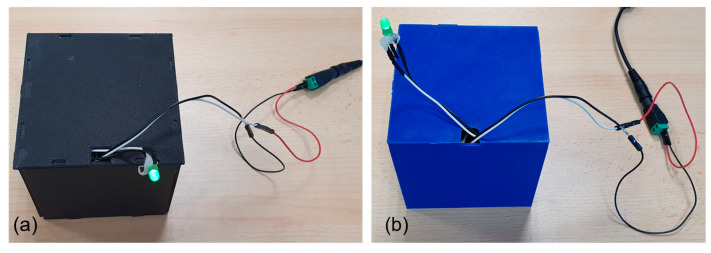
(**a**) Black case realized by reinforced ONYX. (**b**) Blue case realized by PLA.

In addition, with the aim to evaluate significant differences in terms of temperature and measurement output, a comparison between the case structure made by reinforced ONYX and PLA was evaluated. For this purpose, a second structure was made of PLA (Figure 10b), geometrically identical to the one made of reinforced ONYX (Figure 10a); both are shown in Figure 10.

**Figure 11:**
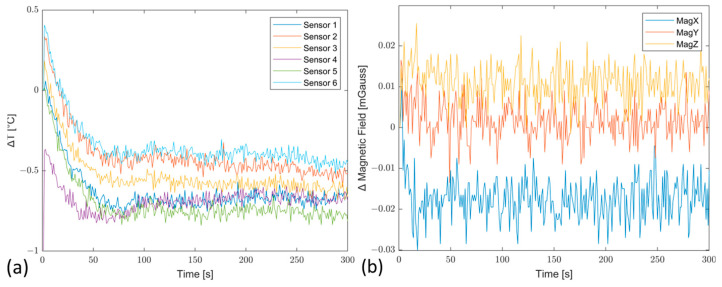
Temperature differences of each sensor (**a**) and average magnetic field differences (**b**) between the case manufactured by reinforced ONYX and PLA.

The results obtained are shown in Figure 11 as the difference between the temperature, ΔT (Figure 11a), and magnetic field values, Δ Magnetic Field (Figure 11b), along the three axes between the reinforced ONYX and PLA structure, showing that apart from the geometric shape, the behavior of the sensors remains unchanged, obtaining significant advantages in terms of material quality and strength.

**Figure 12:**
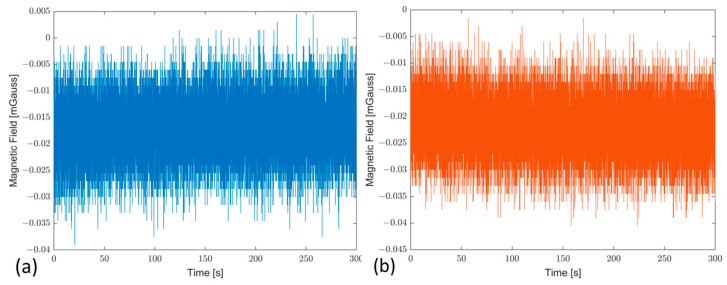
Magnetic field comparison evaluated as differences between the prototype and reference system, referred to as X-axis in blue color (**a**) and Y-axis in red color (**b**).

Finally, the effect of the material on magnetic field measurements was evaluated; the measurements obtained from the prototype and certified AHRS, i.e., Axitude^TM^, are compared to observe any variations in the magnetic field measurement along the x and y components, which are typically used in navigation to assess vehicle orientation by measuring the components of the Earth’s magnetic field. The results obtained are shown in Figure 12a,b for the magnetic field component along the x-axis and y-axis, respectively. The results are evaluated as the difference between the reference system and prototype for a total of 300 s that pointed out average difference values of 0.018 mGauss and 0.02 mGauss for the x-axis and y-axis, respectively. This way, the realized support granted better mechanical characteristics without detriments, with respect to both the previous version of the redundant configuration [24] and the reference systems.

### 3.3. Attitude Estimates

**Figure 13:**
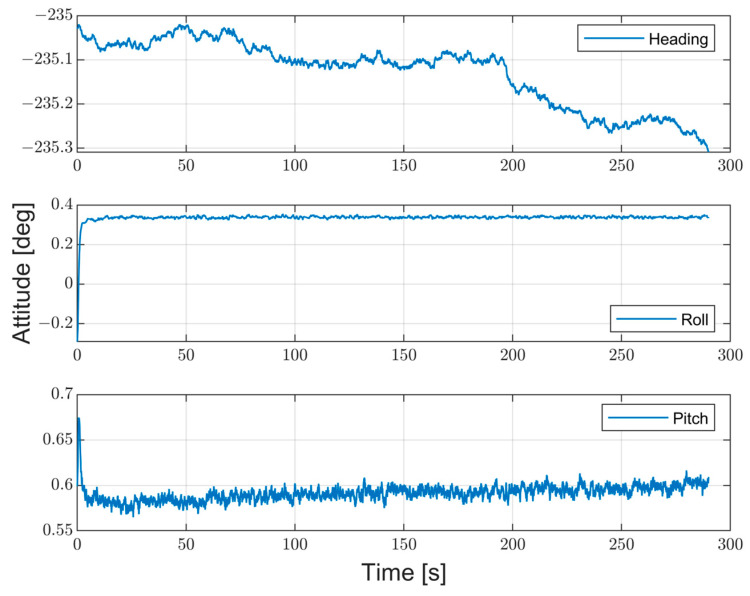
Prototype attitude estimates evaluated in stationary condition.

**Figure 14:**
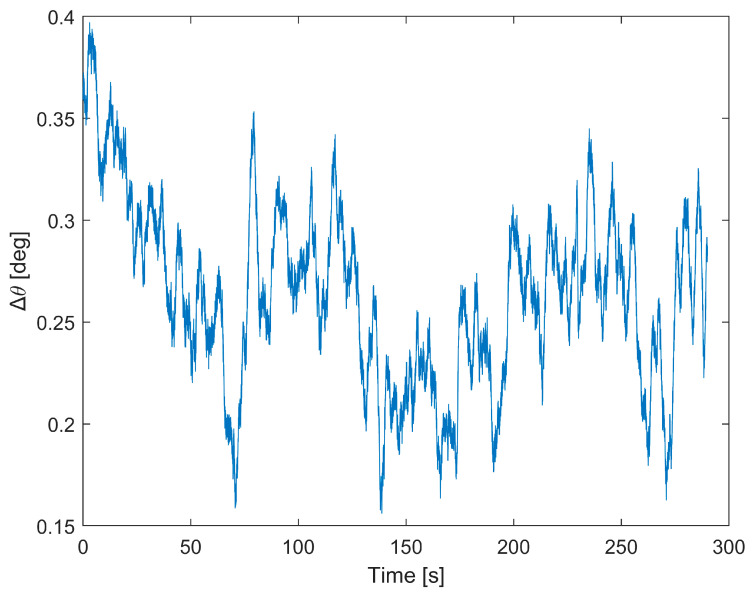
Heading differences between the prototype and reference in static conditions.

The aim of the test is to evaluate the prototype performance in terms of attitude estimations and system stability over time in two different conditions: (i) in stationary conditions and (ii) in dynamic conditions by means of a flight test. The performance is evaluated as RMSE, mean value, and STD of the differences between the reference and the developed system. To this purpose, an Error-State Kalman Filter (ESKF) was adopted to estimate the system attitude, i.e., heading, pitch, and roll angles. The ESKF is already presented in [24,35], but in this application, the ESKF input was the fused measurements according to the proposed method. The first test is conducted in stationary conditions for a time interval of five minutes. Figure 13 shows the prototype attitude estimates, while in Figure 14, the difference between the two systems is highlighted in terms of heading differences (Δ*θ*).

**Figure 15:**
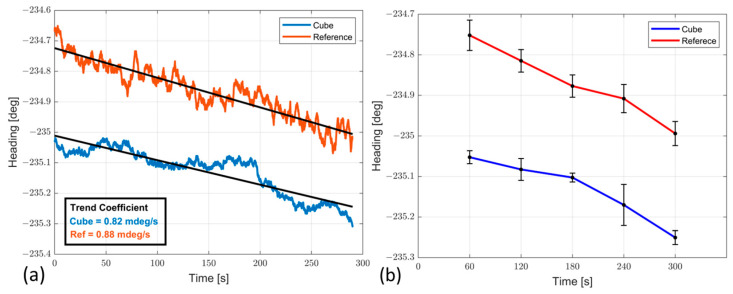
(**a**) Heading angle trend comparison between prototype and reference system; (**b**) error evaluation of both systems in terms of mean and standard deviation.

The RMSE and standard deviation of the heading difference values are equal to 0.26 deg and 0.04 deg, respectively, clearly showing that the values obtained for the headings are consistent with the certified reference system, and this can be further illustrated by Figure 15, where both heading angle trends are evaluated. This figure clearly shows that the certified reference system and the proposed prototype have the same heading angle trend, with coefficient values of 0.88 and 0.82 mdeg/s, respectively (Figure 15a). Finally, the mean value and the standard deviation are obtained as the average of one minute and are shown in Figure 15b, with the associated mean value and standard deviation, while the numerical results are reported in Table 6.

The results in static conditions showed that the attitude estimates differences present a value in RMSE equal to 0.22 deg with the same trend coefficient for both systems and equal to 0.8 mdeg/s, mainly due to not completely compensated Earth rotation contributions.

**Figure 16:**
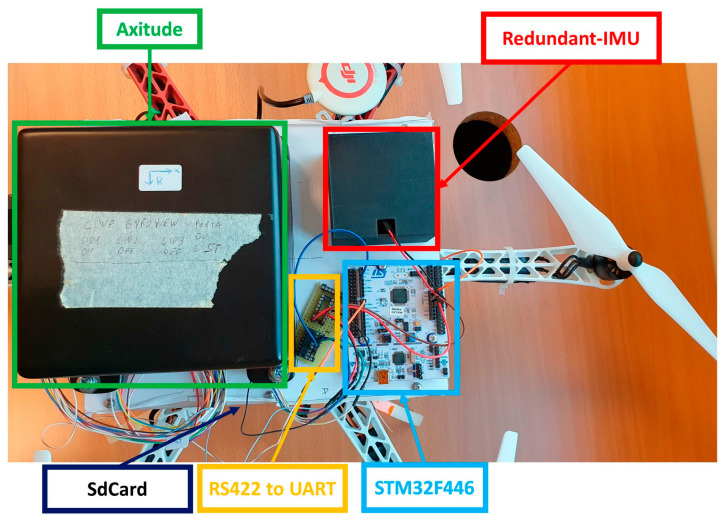
Measurement setup for drone flight test.

**Figure 17:**
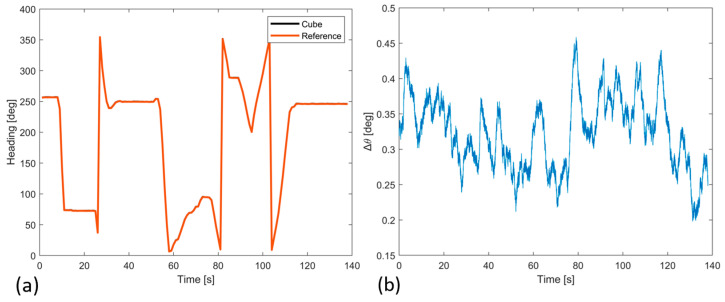
(**a**) Heading angle of the redundant-IMU (black) and reference (red); (**b**) heading differences between the prototype and reference in dynamic conditions.

Finally, dynamic tests were conducted to assess the prototype performance by means of a drone flight where arbitrary manual rotations were carried out in both high-rate and low-rate conditions. In particular, the measures of the prototype (with enclosure) and certified AHRS are acquired with a microcontroller and then stored in a microSD-Card (lower level of drone), as shown in Figure 16. The results are shown in terms of heading estimation in Figure 17a as a comparison between both systems that are fully overlapped for the total flight, and in Figure 17b, where the differences are highlighted to better appreciate the comparison of both systems. The attitude results, i.e., heading, pitch, and roll angles, are reported in Table 7, which are evaluated as RMSE, mean value, and STD of the difference between the prototype and the reference. For both tests (static and dynamic), the estimated heading angle is shown in the figures, which is not only the most difficult to estimate due to the Earth’s rotations and the initial unknown north direction but also is the reference for the aeronautical standard, which is expected to have an accuracy of ± 3 degrees.

Thanks to the enhanced methods adopted, the systems showed comparable performance with the certified reference system, and the error analysis evaluated makes the measurements compliant with the reference system in both tests, i.e., static and dynamic.

## 4. Conclusions

In this research, we proposed a novel approach to fuse raw measurements from a redundant IMU that exploits the capabilities of reinforced material with Carbon Fiber. In particular, the results of the Allan variance evidenced that, despite all the same sensors present, different trends correspond to various noise parameters. Nevertheless, the innovative approach of the weighted average at each sensor allowed us to enhance the benefits due to the redundancy associating the correct impact on the final fused raw measures of multi-sensor platforms, where the evaluation of weights is obtained according to the noise parameter value evaluation. A performance evaluation between raw measurements of the reference system and the enhanced redundant-IMU exhibited an improvement in the overall measures evaluated as differences in terms of RMSE, mean value, and standard deviation. Moreover, a PDF comparison between the raw measurements, evaluated as weighed average and average value, was analyzed; the results highlight a Gaussian trend improvement that is suitable for a navigation system based on the Kalman Filter algorithm.

The introduction of innovative material, i.e., reinforced ONYX, as a structure and case for the navigation system led to investigating the effects on the measures, where two critical aspects, such as internal temperature and magnetic field measurements, were evaluated. The results carried out in both analyses pointed out that the material has no significant effect, as shown by the comparison with PLA material and also by the comparison of magnetic field measurements with the certified system. Therefore, thanks to additive manufacturing, the realized prototype maintained a suitable shape factor for the Unmanned Aerial System, such as a drone, keeping advantageous mechanical properties thanks to the custom design and the innovative material adopted.

Finally, to assess the overall performance of the proposed solution, the attitude estimates, i.e., heading, pitch, and roll angles, showed remarkable performance that is evaluated as a comparison between the prototype and reference system, where the results highlighted differences of about 0.22 deg and the same angle trend with respect to the reference system in a static test, while to evaluate the system in dynamic conditions, a drone flight test was performed, where the attitude estimate results are evaluated as differences between the two systems and values of 0.32 deg were observed.

## Figures and Tables

**Table 1:** Bias Instability (BI) and Velocity Random Walk (VRW) of the multi-sensor redundant platform accelerometer.

Accelerometer	BI [mg]	$\mathrm{VRW}\;[m/s/\sqrt{h}]$
x-axis	0.02	0.01
y-axis	0.02	0.02
z-axis	0.03	0.01

**Table 2:** Bias Instability (BI) and Angular Random Walk (ARW) of the multi-sensor redundant platform gyroscope.

Gyroscope	BI [deg/h]	$\mathrm{ARW}\;[\deg/\sqrt{h}]$
x-axis	3.1	0.11
y-axis	3.1	0.11
z-axis	1.8	0.12

**Table 3:** Bias Instability (BI), Angular Random Walk (ARW), and Velocity Random Walk (VRW) of the STIM300 tactical-grade as reference sensor.

	Accelerometer	Gyroscope
BI	0.04 [mg]	0.3 [deg/h]
V/A RW	0.07 [m/s/$\sqrt{h}$]	0.15 [deg/$\sqrt{h}$]

**Table 4:** Allan variance (AV) results obtained with the proposed approach from the redundant-IMU.

Prototype AV Results	Accelerometer		Gyroscope	
	BI [mg]	VRW [m/s/$\sqrt{h}$]	BI [deg/h]	$\mathrm{ARW}\;[\deg/\sqrt{h}]$
x-axis	0.001	0.003	0.14	0.013
y-axis	0.001	0.003	0.15	0.024
z-axis	0.002	0.002	0.14	0.021

**Table 5:** Performance comparison as the difference between the raw measurements of the reference system and proposed method.

	RMSE	Mean Value	STD
**Accelerometers [g]**			
z-axis	0.0121	0.0118	0.0021
y-axis	0.0231	0.0231	0.0022
x-axis	0.0129	0.0127	0.0024
**Gyroscopes [deg/s]**			
z-axis	0.0839	0.0391	0.0742
y-axis	0.0801	0.0096	0.0795
x-axis	0.0823	0.0145	0.0811

**Table 6:** Results obtained from the prototype and reference system evaluated as mean value, standard deviation, and differences Δ*θ* between both systems.

	Prototype		Reference		Δ*θ*
Time [s]	Mean Value [deg]	STD [deg]	Mean Value [deg]	STD [deg]	[deg]
60	235.053	0.015	234.752	0.037	0.3
120	235.083	0.026	234.815	0.027	0.267
180	235.103	0.011	234.877	0.027	0.225
240	235.17	0.05	234.908	0.034	0.262
300	235.251	0.017	234.994	0.029	0.256

**Table 7:** Attitude results in dynamic conditions.

Attitude Differences	RMSE [deg]	Mean Value [deg]	STD [deg]
Heading	0.327	0.323	0.051
Pitch	0.175	0.152	0.085
Roll	0.171	0.149	0.079

## Data Availability

Not applicable.
